# Exploring the Metabolic Vulnerabilities of Epithelial–Mesenchymal Transition in Breast Cancer

**DOI:** 10.3389/fcell.2020.00655

**Published:** 2020-07-24

**Authors:** Xiangyu Sun, Mozhi Wang, Mengshen Wang, Litong Yao, Xinyan Li, Haoran Dong, Meng Li, Xiang Li, Xing Liu, Yingying Xu

**Affiliations:** Department of Breast Surgery, The First Affiliated Hospital of China Medical University, Shenyang, China

**Keywords:** breast neoplasms, epithelial–mesenchymal transition, metabolic reprogramming, glycolysis, metastasis

## Abstract

Metastasis and drug resistance are the leading causes of death for breast cancer patients. Epithelial–mesenchymal transition (EMT), a transition from polarized epithelial cells to motile mesenchymal cells mediated by a series of activation signals, confers breast tumor cells with enhanced stem cell, invasive, and metastatic properties. Metabolic reprogramming is an emerging hallmark of cancer cells, which have a complex mutual effect with EMT process. Under hypoxic and nutrient-deprived conditions, metabolic rewiring can rapidly provide ATP and sufficient metabolic intermediates for fueling breast cancer metastasis and progression. In this review, we primarily focus on how these altered metabolic phenotypes of breast tumor cells activate the EMT transcription factors and induce the EMT process to further promote metastasis and resistance to therapy. This review is divided to glucose, lipid, and amino acid metabolism to explore for potential metabolic vulnerabilities, which may provide new insights for blocking the EMT process in breast cancer.

## Introduction

Breast cancer is the most common cancer in female individuals (Fan et al., 2014; Siegel et al., 2019). Although advanced diagnostic and treating approaches have been developed, metastasis and drug resistance are still the leading causes of death from breast cancer (Eckhardt et al., 2012). Breast cancer is classified into four molecular subtypes, namely, luminal A, luminal B, human epidermal growth factor receptor 2 (HER2)-positive, and triple-negative breast cancer (TNBC) according to the status of the estrogen receptor, progesterone receptor, and HER2 (Perou et al., 2000). The epithelial–mesenchymal transition (EMT) process is characterized by a transition from polarized epithelial cells to motile mesenchymal cells mediated by a series of biological events, endowing breast tumor cells stem cell, invasive, and metastatic properties (Foroni et al., 2012; Yeung and Yang, 2017). Notably, EMT is accompanied with altered metabolic phenotypes, especially in glucose, lipid, and amino acid metabolic pathways. These metabolic adaptions enable breast tumor cells to express EMT transcription factors (EMT-TFs), transform from epithelial cancer cells to mesenchymal types, and adopt breast cancer stem cell (BCSC)-like properties to fuel cancer stemness and metastasis (Sciacovelli and Frezza, 2017). Thus, understanding the causes and the consequences of altered metabolism during the EMT process may permit the identification of drug targets for treating breast cancer. In this review, we primarily target the crucial metabolic pathways, including glucose, lipid, and amino acid, to explore the metabolic vulnerabilities involved in the EMT initiation and development in breast cancer.

## The Significance of the EMT Process in Breast Cancer

Epithelial–mesenchymal transition confers breast tumor cells increased tumor-initiating and metastatic potential (V. Mittal, 2018; Pastushenko and Blanpain, 2019). The EMT process is characterized by the upregulation of mesenchymal markers, including N-cadherin and vimentin, and the reduction of epithelial marker E-cadherin (Wu et al., 2016). EMT is regulated by multiple EMT-TFs, primarily Twist-related protein (Twist), Snail, zinc finger E-box binding homeobox (ZEB), and several regulatory molecules (Yang et al., 2004; Felipe Lima et al., 2016). Mechanistically, these EMT-TFs further induce the altered expression and activity of downstream signaling involved in stemness, invasion, and metastasis. Thus, blocking the activation of these EMT-TFs is the primary focus for suspending the invasive and the metastatic potentials of breast cancer.

It is well established that the EMT process endows breast tumor cells with mesenchymal properties to generate greater resistance to elimination by multiple therapeutic approaches. EMT confers breast tumor cells with BCSC-like features to acquire resistance to therapies, which may be associated with the regulation of genes involved in cell survival, stem cell maintenance, and therapy resistance-related genes (Mani et al., 2008; Fischer et al., 2015; van Staalduinen et al., 2018). For instance, mesenchymal breast tumor cells display prominent resistance to antitumor immune response. Mammary mesenchymal cancer cells were less susceptible to immunotherapy than corresponding epithelial tumors since mesenchymal cells are characterized by the high expression of PD-L1 and the low levels of MHC-I (Dongre et al., 2017). EMT also impairs the susceptibility of breast tumor cells to immune surveillance mediated by T cells (Akalay et al., 2013a, b). Additionally, EMT is required for the generation of chemotherapy resistance in breast tumor cells. It was shown that breast tumor cells undergoing EMT can survive cyclophosphamide treatment due to impaired apoptotic tolerance and the elevated expression of chemoresistance-related genes (Fischer et al., 2015). Therefore, EMT orchestrates both metastasis and therapy resistance for breast cancer progression, indicating a crucial role of EMT in breast cancer progression.

**FIGURE 1:**
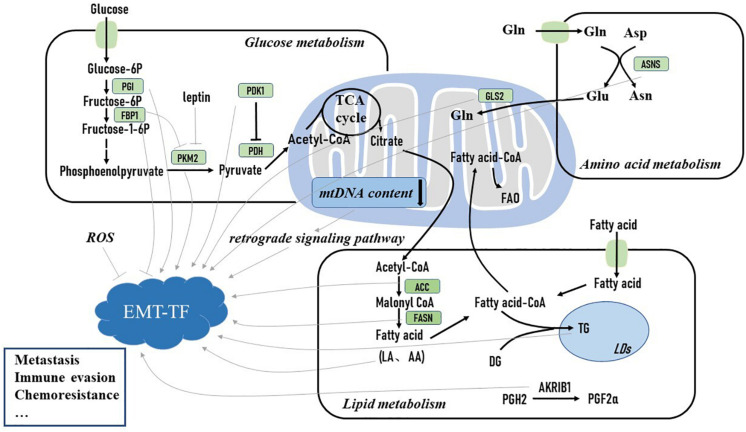
Metabolic reprogramming activates epithelial–mesenchymal transition (EMT)-inducing transcription factor and induces the EMT process to promote breast cancer metastasis and drug resistance. Glucose-6P, glucose 6-phosphate; fructose-6P, fructose 6-phosphate; fructose-1-6P, fructose-1-6-phosphate; PGI, phosphoglucose isomerase; FBP1, fructose-1,6-bisphosphatase 1; PKM2, pyruvate kinase M2; PDH, pyruvate dehydrogenase; PDK1, pyruvate dehydrogenase kinase 1; TCA cycle, tricarboxylic acid cycle; mt DNA, mitochondrial DNA; FAO, fatty acid oxidation; Gln, glutamine; GLS2, glutaminase 2; DG, diglyceride; TG, triglyceride; LA, linoleic acid; AA, arachidonic acid; PGH2, prostaglandin H2; PGF2α, prostaglandin F2α; LDs, lipid droplets; ACC, acetyl-coA carboxylase; FASN, fatty acid synthase; AKR1B1, aldo-keto reductase 1 member B1; Glu, glutamate; Asp, aspartate; Asn, asparagine; ASNS, asparagine synthetase; ROS, reactive oxidative stress; EMT-TF, epithelial–mesenchymal transition transcription factor.

Substantial studies have illustrated that EMT has a complex mutual relationship with metabolic reprogramming in cancer cells (Sciacovelli and Frezza, 2017). Metabolic reprogramming is an emerging hallmark of cancer cells. During rapid proliferation, cancer cells have to alter their metabolic phenotype to produce sufficient ATP and important intermediates to sustain their survival (Zhu and Thompson, 2019). EMT is accompanied by complex metabolic rewiring (Huang and Zong, 2017). During the EMT process, metabolic reprogramming is required for breast tumor cells to support their energy demand of increased motility and invasion in nutrient-deprived and hypoxic environmental conditions (Sciacovelli and Frezza, 2017), yet how EMT regulates metabolic adaptation is still poorly understood. Shaul et al. analyzed the metabolic gene expression in a set of high-grade cancer cell lines expressing mesenchymal markers and identified several mesenchymal metabolic signature genes. Upon EMT induction, these genes were found to be upregulated in human mammary epithelial cells (Shaul et al., 2014). This finding suggests that the EMT program may directly influence the expression of metabolic genes. Moreover, how the dysregulated metabolic pathways regulate EMT initiation and development are under more substantial studies (Figure 1). Herein we primarily focus on how metabolic pathways activate EMT-TFs and induce the EMT process to further promote breast cancer progression.

## Metabolic Rewiring Induces the EMT Process in Breast Cancer

### Glucose Metabolism

Cancer cells are characterized by the metabolic shift from mitochondrial oxidative metabolism to aerobic glycolysis to rapidly produce sufficient energy and important intermediates, which is crucial for the increased invasive and metastatic potential (Gatenby and Gillies, 2004). EMT induction is accompanied with enhanced aerobic glycolysis along with the overexpression of crucial glycolysis-related enzymes in breast cancer (Kondaveeti et al., 2015). For instance, pyruvate kinase M2 (PKM2), participating in the final rate-limiting step of glycolysis, is significantly associated with the EMT process. Fructose-1,6-biphosphatase (FBP1) suppresses PKM2 activation to abrogate glycolysis, while it also increases mitochondrial complex I activity to enhance oxidative phosphorylation (OXPHOS). Thus, loss of FBP1 is essential to increase glycolytic intermediates for biosynthesis and sustain ATP production, which leads to increased BCSC-like properties for Snail-mediated EMT (Dong et al., 2013). The signals from the microenvironment may also alter the expression of PKM2 and reprogram the glycolytic phenotype of breast cancer to induce the EMT process. The adipocyte-derived leptin, a crucial adipokine in fueling breast cancer progression, promotes EMT *via* the upregulation of PKM2 expression and the activation of PI3K/AKT signaling pathway (Wei et al., 2016). Recently, the phosphorylation of PKM2 was described to promote BCSC-like cell properties *via* the activation of yes-associated protein (YAP) downstream signaling. PKM2 is phosphorylated at tyrosine 105 by activated kinases, which confers PKM2 oncogenic function in breast cancer cells *via* promoting YAP nuclear translocation. YAP silencing suspends oncogenic kinase-induced BCSC properties to block the EMT process and reverses resistance to chemotherapy (Li et al., 2018; Zhou et al., 2018). Overall, these findings suggest that PKM2 and its downstream signaling may be effective targets to reverse the mesenchymal phenotype.

Pyruvate dehydrogenase kinase 1 (PDK1) is another crucial glycolytic enzyme and inactivates the pyruvate dehydrogenase complex that allows pyruvate to undergo tricarboxylic acid (TCA) cycle. PDK1 is necessary for the formation of liver metastases *via* the promotion of glycolytic metabolism (Dupuy et al., 2015). Moreover, PDK1 is required for EMT induction. PDK1 inhibition effectively downregulates mesenchymal markers and suspends lung-specific metastatic potential. The exogenous expression of PDK1 allows PDK1-silencing breast tumor cells to regain mesenchymal properties (Du et al., 2016). Furthermore, a long non-coding RNA H19, which is significantly associated with PDK1 expression, is crucial for glycolytic activity and BCSC properties. H19 silencing abrogates PDK1 expression under hypoxia, glycolysis, and self-renewal capability. Importantly, aspirin can markedly attenuate BCSC properties by inhibiting both H19 and PDK1, which may add new insights for blocking the EMT process (Peng et al., 2018).

Phosphoglucose isomerase (PGI), catalyzing the interconversion of glucose 6-phosphate and fructose 6-phosphate, regulates EMT in the initial stage of cancer metastasis and mesenchymal–epithelial transition in the final stage of metastasis during breast cancer colonization. Specifically, PGI/AMF over-expression induces EMT in normal mammary epithelial cells, allowing the escape of these cells from the primary tumor. Furthermore, inhibiting PGI/AMF expression contributes to mesenchymal–epithelial transition in aggressive breast tumor cells, assisting their colonization and growth at secondary sites (Funasaka et al., 2009). A follow-up study found that PGI/AMF over-expression promotes EMT through enhancing the DNA binding activity of nuclear factor κB (NF-κB) and further transcriptionally upregulating the expression of ZEB. ZEB expression can be negatively regulated by microRNA-200s, suggesting miR-200s as a therapeutic target in blocking PGI/AMF-induced EMT (Ahmad et al., 2011).

Cancer cells may undergo metabolic switching from OXPHOS to glycolysis in hypoxia during rapid proliferation (Avagliano et al., 2019). Thus, declined OXPHOS activity has been commonly described in breast tumor cells. Declined OXPHOS activity may be the consequence of mitochondrial DNA (mtDNA) mutation or less mtDNA content, coding for the OXPHOS protein complexes (Ashton et al., 2018). Reduced mtDNA content promotes a calcineurin-dependent mitochondrial retrograde signaling pathway, which induces the EMT process and BCSC properties (Guha et al., 2014). The role of reduced mtDNA content and declined OXPHOS activity in EMT induction may potentially provide novel targets for targeting metastasis.

Oxidative stress also plays a critical role in EMT induction. One emerging idea is that the reduction of mitochondrial-derived reactive oxygen species (ROS) may induce the EMT process. Despite that both BCSC states display upregulated glycolysis-related genes, mesenchymal and epithelial BCSCs make the response to oxidant stress *via* distinct metabolic pathways and redox potential. In this scenario, elevated ROS induces mesenchymal BCSCs to transform to the epithelial state. Thus, mesenchymal BCSCs are characterized by declined OXPHOS potential and low levels of ROS (Luo et al., 2018a). NADH and NADPH are the primary sources of reducing equivalent participation in ROS detoxification, thereby serving as key factors in suppressing intracellular ROS (Ying, 2008). Thus, the NAD(P)H level may interconnect ROS and the EMT process. Overexpression of NAD(P)H:quinone oxidoreductase-1 (NQO1) regulates the expression of pyruvate kinase in the liver and the red blood cells (PKLR) to mediate EMT in breast cancer. NQO1 bounds to PKLR to enhance the glycolytic phenotype *via* maintaining NAD(P)H homeostasis. NQO1 silencing remarkably increases intracellular ROS, which could inhibit the EMT process (Yang et al., 2019). Thus, the NQO1/PKLR network supports the EMT induction and may be an effective therapeutic target for blocking the EMT process. Consistently, C-terminal binding protein (CtBP), a key downstream epigenetic effector of elevated NAD(P)H, was shown to induce mesenchymal and BCSC features in breast cancer cells (Di et al., 2013). CtBP inhibition significantly blocks the EMT process, identifying CtBP as a pharmacologic target for suspending EMT. Nevertheless, there are conflicting viewpoints that elevated ROS level may also accompany EMT and BCSC-like properties in breast cancer (Feng et al., 2018; Xiong et al., 2018). Elevated matrix metalloproteinase-3, a signal from the breast tumor microenvironment, increases the level of ROS in breast tumor cells. The elevated ROS further induces the expression of Snail and EMT (Radisky et al., 2005). Consistently, it was shown that enhanced mitochondrial OXPHOS and elevated ROS level may get involved in the maintenance of BCSCs in TNBC (Lee et al., 2017). Further studies are needed to illustrate the precise role of ROS in mediating EMT induction in breast cancer.

### Lipid Metabolism

In addition to the crucial role of glucose metabolism in EMT induction, aberrant lipid metabolism is also involved in the development of EMT in breast cancer. Previous studies have revealed that *de novo* lipogenesis is enhanced by oncogenic signaling in breast tumor cells for the generation of sufficient membrane phospholipids and signal molecules to prepare for invasion and metastasis. Sterol regulatory element-binding transcription protein 1 (SREBP1), the master transcriptional regulator of lipogenesis, can enhance *de novo* lipogenesis *via* reinforcing the expression of key lipogenic genes. SREBP1 acts as an EMT regulator *via* forming a co-repressor complex with Snail and histone deacetylase to suppress E-cadherin in breast cancer. In this regard, SREBP1 inhibition could be mediated by miR-18a-5p, which inhibits the following EMT process and lung metastasis of breast tumors (Zhang et al., 2019). Additionally, fatty acid synthase (FASN), a critical lipogenic enzyme, is essential for EMT development in breast cancer. Cerulenin, a FASN inhibitor, could significantly impair the EMT process (Li et al., 2014). Furthermore, hyperglycemia-induced EMT phenotype can also be reversed upon FASN inhibition (Zielinska et al., 2018). Nevertheless, FASN silencing was found to be sufficient to induce transforming growth factor beta 1 (TGFβ1)-induced EMT and metastasis (Jiang et al., 2015). These conflicting results need further investigation. Acetyl-CoA carboxylase1 (ACC1), another crucial lipogenic enzyme, is a pivotal player in the EMT process of breast cancer. It is not only capable of promoting *de novo* lipogenic pathway by converting acetyl-CoA to malonyl-CoA but also involved in protein acetylation. A recent study indicates that ACC1 may regulate the EMT process through the latter function. ACC1 inhibition elevates acetyl-CoA level, and this further induces Smad2 acetylation and the EMT process. Notably, the activation of leptin or TGFβ signaling, which is widely observed in breast cancer patients with obesity, presents with inhibited ACC1 expression to promote the initiation of EMT (Rios Garcia et al., 2017). Thus, targeting the ACC1-dependent EMT axis may provide an effective strategy for the treatment of breast cancer patients with obesity.

Accumulating studies suggest a series of lipid-related metabolic enzymes that may serve as therapeutic targets for blocking the EMT process in breast cancer. For instance, aldo-keto reductase 1 member B1 (AKR1B1), which converts prostaglandin H2 to prostaglandin F2α, is a key enzyme in arachidonic acid metabolism. Twist transcriptionally upregulates AKR1B1 expression and further activates NF-κB. Subsequently, NF-κB promotes Twist expression and forms a positive feedback loop that induces the EMT program and enhances BCSC-like properties. Epalrestat, an AKR1B1 inhibitor, can significantly suppress the EMT process. Thus, AKR1B1 may be a potential therapeutic target for TNBC (Wu et al., 2017). Besides that, lipid-transfer protein (Nir2) serves as a novel regulator of EMT in breast cancer cells. Nir2 silencing attenuates TGFβ1-induced EMT, making Nir a valuable therapeutic target (Keinan et al., 2019). Ganglioside 2 (GD2) is identified as a new marker for BCSCs since GD2-positive breast tumor cells exhibit BCSC-like features (Battula et al., 2012). GD3 synthase (GD3S), the rate-limiting enzyme for GD2 biosynthesis, is required for the induction and the development of EMT. EMT induction significantly elevates GD2 content and GD3S expression in breast tumor cells (Sarkar et al., 2015). Thus, inhibiting GD3S may provide new insights to conquer EMT of breast cancer.

In the comparison of proteomic and lipidomic results between epithelial and mesenchymal breast tumor cells, mesenchymal breast tumor cells are characterized by an increased expression of genes related to triglyceride synthesis and lipid droplet formation, while epithelial breast cancer cells are featured as the upregulated expression of genes involved in *de novo* fatty acid synthesis. Thus, targeting triglyceride metabolism may be an effective therapeutic approach for blocking EMT (Giudetti et al., 2019). In order to decipher the lipid composition of breast tumor cells undergoing EMT, increased levels of phosphatidylcholine and triacylglycerol, as well as a decreased level of diacylglycerol, were found to follow the EMT process (Eiriksson et al., 2018). Further studies are required to explore the alterations of lipid types accumulated in breast cancer cells undergoing EMT, which are beneficial for the understanding of the relationship between lipid alteration and the EMT process. Exogenous fatty acids, such as linoleic acid and arachidonic acid, were found to initiate EMT in MCF10A (Martinez-Orozco et al., 2010; Espinosa-Neira et al., 2011). These observations provoke thoughts that the limitation of exogenous fatty acid uptake may present therapeutic opportunities for exploring EMT induction.

### Amino Acid Metabolism

Amino acid metabolism plays a critical role in aggressiveness, invasion, and metastasis of breast cancer (Bertero et al., 2019). Recent studies suggest that several key enzymes related to amino acid metabolism are significantly upregulated in breast cancer tissues and associated with high metastatic properties. Nevertheless, the role of altered amino acid metabolism in modulating EMT has not been clearly defined in breast cancer. Metabolomic profiling of a breast epithelial cell line and its EMT-derived mesenchymal phenotype illustrates that the mesenchymal phenotype is more addicted to amino acid anaplerosis, while the epithelial phenotype shows higher addiction to glycolysis and OXPHOS (Halldorsson et al., 2017). This demonstrates the importance of amino acid metabolism in promoting the EMT process.

Asparagine acts as a powerful regulator in driving the EMT process of breast cancer. The level of asparagine is specifically elevated in proteins driving EMT. Moreover, limiting asparagine bioavailability was found to attenuate the expression of proteins driving EMT. Asparagine synthetase, which modulates asparagine biosynthesis, may be a feasible therapeutic target to reduce asparagine content and therefore block the EMT process to impair the invasive and metastatic potential of breast cancer. Besides that, limiting asparagine by treatment with L-asparaginase or dietary asparagine also abrogates EMT induction to reduce breast cancer metastasis (Knott et al., 2018; Luo et al., 2018b).

Cystine addiction is a key metabolic characteristic of TNBC cells, while luminal breast cancer cells are thought to be cystine independent. Based on this, cystine deprivation leads to rapid programmed necrosis in cystine-addictive TNBC phenotype and little death in luminal-type breast cells (Tang et al., 2017). Moreover, this cystine-addictive phenotype is suspended in these cystine-addictive cells *via* the introduction of miR-200c, which reverses mesenchymal features. Thus, although the underlying mechanisms are not clear, this specific cystine-addictive phenotype of TNBC is dramatically associated with EMT. Cystine addiction may be a research hotspot in the TNBC associated with EMT.

Glutamine is the most abundant amino acid. TNBC highly depends on glutamine metabolism for progression, which is primarily mediated by glutaminase isoforms (Timmerman et al., 2013). Glutaminase 2 (GLS2) was found to increase mesenchymal markers, invasion, and metastasis (Dias et al., 2020). Conflictingly, another study suggests that the levels of GLS2 are inversely correlated with EMT. A comparison of the metabolic phenotype and the gene expression between mesenchymal and epithelial breast tumor cells illustrates that the loss of GLS2 expression upon the induction of EMT leads to enhanced glutamine-independent phenotype and reduced mitochondrial activity, while the restoration of GLS2 expression in GLS2-negative breast cancer cells increases mitochondrial glutamine consumption and suspends BCSC-like properties (Ramirez-Peña et al., 2019). These opposing results need further explanations.

## Conclusion

The EMT process endows breast tumor cells with stem cell, invasive, and migratory properties, which contribute to enhanced metastasis and drug resistance. Metabolic reprogramming, as a key feature of cancer cells, is considered to have a mutual effect with the EMT process. As discussed above, we primarily focused on how metabolic rewiring induces the EMT process to promote breast cancer progression. The altered metabolic phenotype of breast tumor cells, especially glucose, lipid, and amino acid metabolism, may regulate the expression of EMT-TFs to induce the EMT process. However, it should be noted that most metabolic pathways are interdependent and closely connected. For instance, large amounts of glucose are taken up by cancer cells, but most of this carbon is excreted as lactate rather than metabolized in the TCA cycle. In this scenario, glutamine metabolism can serve as an alternative source of carbon to the TCA cycle for citrate production and fatty acid synthesis (Altman et al., 2016). The cross-talks between these metabolic pathways are well illustrated by the fact that master genes, such as p53 and MYC, regulate these metabolic pathways in a coordinated manner (Stine et al., 2015; Flöter et al., 2017). In addition, metabolic adaptation should not only be focused in the context of EMT itself; it may also be involved in the stabilization of the mesenchymal phenotype of breast cancer. Selective targeting of these metabolic vulnerabilities may provide new insights to block EMT induction and therefore prevent EMT-related metastasis and drug resistance. Nevertheless, the interplay between EMT and metabolic reprogramming is far more complicated, and the causes and the consequences of EMT development need more extensive studies.

## Author Contributions

XS contributed to writing and elaborating the figures. MoW, MeW, LY, XinL, HD, ML, XiaL, and XYL contributed to writing and reviewing the manuscript. YX contributed to writing and reviewing the final version. All authors contributed to the article and approved the submitted version.

## Conflict of Interest

The authors declare that the research was conducted in the absence of any commercial or financial relationships that could be construed as a potential conflict of interest.
